# Prof Stephen P. Long, FRS (1950–2025)

**DOI:** 10.1111/gcb.70606

**Published:** 2025-12-10

**Authors:** Lorenzo Álvarez‐Filip, Edith Bai, Carl J. Bernacchi, Rhea Bruno, Klaus Butterbach‐Bahl, Maria Byrne, I‐Ching Chen, Shaolin Chen, William W. L. Cheung, M. Francesca Cotrufo, Tatenda Dalu, Xiaojuan Feng, Yongshuo Fu, Yanli Gao, Vera L. M. Huszar, Ivan A. Janssens, Sujong Jeong, T. Hefin Jones, Stephen D. Joseph, Madhu Khanna, Miko U. F. Kirschbaum, Kazuhiko Kobayashi, Julie LaRoche, Andrew D. B. Leakey, Xinhai Li, Yin Li, Lingli Liu, Annalea Lohila, Yiqi Luo, Andrew E. McKechnie, Ara Monadjem, Rachael H. Nolan, Richard G. Pearson, Shushi Peng, Josep Peñuelas, Shilong Piao, Sharon A. Robinson, Youngryel Ryu, Rowan F. Sage, Rachel G. Shekar, Shuai Xue, Pete Smith, Glaucia Mendes Souza, Sabrina Spatari, David J. Suggett, Guangce Wang, Danielle A. Way, Jin Wu, Longlong Xia

**Affiliations:** ^1^ Biodiversity and Reef Conservation Laboratory, Unidad Académica de Sistemas Arrecifales Instituto de Ciencias del Mar y Limnología, Universidad Nacional Autónoma de México Puerto Morelos Mexico; ^2^ Key Laboratory of Geographical Processes and Ecological Security of Changbai Mountains, Ministry of Education, School of Geographical Sciences Northeast Normal University Changchun Jilin China; ^3^ Key Laboratory of Vegetation Ecology, Ministry of Education Northeast Normal University Changchun Jilin China; ^4^ Department of Crop Sciences University of Illinois Urbana‐Champaign Urbana Illinois USA; ^5^ Carl R. Woese Institute for Genomic Biology University of Illinois at Urbana‐Champaign Urbana Illinois USA; ^6^ Department of Agroecology Aarhus University Tjele Denmark; ^7^ Land‐CRAFT, Department of Agroecology Aarhus University Aarhus Denmark; ^8^ Marine Invertebrate Futures Group, School of Life and Environmental Sciences The University of Sydney Sydney New South Wales Australia; ^9^ Department of Life Sciences National Cheng Kung University Tainan Taiwan; ^10^ College of Life Sciences Northwest A&F University Yangling Shaanxi China; ^11^ Biomass Energy Center for Arid and Semi‐Arid Lands Northwest A&F University Yangling Shaanxi China; ^12^ State Key Laboratory for Crop Stress Resistance and High‐Efficiency Production Northwest A&F University Yangling Shaanxi China; ^13^ Institute for the Oceans and Fisheries University of British Columbia Vancouver British Columbia Canada; ^14^ Department of Soil and Crop Sciences Colorado State University Fort Collins Colorado USA; ^15^ Aquatic Systems Research Group, School of Biology and Environmental Sciences University of Mpumalanga Nelspruit South Africa; ^16^ State Key Laboratory of Forage Breeding‐by‐Design and Utilization, Key Laboratory of Vegetation and Environmental Change Institute of Botany, Chinese Academy of Sciences Beijing China; ^17^ China National Botanical Garden Beijing China; ^18^ College of Resources and Environment University of Chinese Academy of Sciences Beijing China; ^19^ College of Water Sciences Beijing Normal University Beijing China; ^20^ Department of Biology University of Antwerp Wilrijk Belgium; ^21^ Wiley Beijing China; ^22^ Laboratory of Phycology, National Museum Federal University of Rio de Janeiro – UFRJ, Quinta da Boa Vista, São Cristóvão Rio de Janeiro Brazil; ^23^ Department of Environmental Management Seoul National University Seoul South Korea; ^24^ School of Biosciences Cardiff University Cardiff UK; ^25^ School of Materials Science and Engineering University of New South Wales Kensington New South Wales Australia; ^26^ Center for Advanced Bioenergy and Bioproducts Innovation University of Illinois at Urbana‐Champaign Urbana Illinois USA; ^27^ Department of Agriculture and Consumer Economics University of Illinois at Urbana‐Champaign Urbana Illinois USA; ^28^ Bioeconomy Science Institute, Landcare Research Group Lincoln New Zealand; ^29^ Yokoyama Kazuya Cancer Research Institute Tokyo Japan; ^30^ Department of Global Agricultural Sciences, Graduate School of Agricultural and Life Sciences The University of Tokyo Tokyo Japan; ^31^ Department of Biology Dalhousie University Halifax Nova Scotia Canada; ^32^ Institute for Comparative Genomics Dalhousie University Halifax Nova Scotia Canada; ^33^ Department of Plant Biology University of Illinois Champaign Urbana Illinois USA; ^34^ Key Laboratory of Animal Ecology and Conservation Biology Institute of Zoology, Chinese Academy of Sciences Beijing China; ^35^ College of Life Sciences University of Chinese Academy of Sciences Beijing China; ^36^ Department of Microbial Physiological & Metabolic Engineering, State Key Laboratory of Microbial Diversity and Innovative Utilization Institute of Microbiology, Chinese Academy of Sciences Beijing China; ^37^ University of Chinese Academy of Sciences Beijing China; ^38^ Finnish Meteorological Institute Helsinki Finland; ^39^ Institute for Atmospheric and Earth System Research (INAR), Faculty of Sciences University of Helsinki Helsinki Finland; ^40^ Soil and Crop Sciences Section, School of Integrative Plant Science Cornell University Ithaca New York USA; ^41^ Department of Zoology and Entomology University of Pretoria Pretoria South Africa; ^42^ Department of Biological Sciences University of Eswatini Kwaluseni Eswatini; ^43^ Hawkesbury Institute for the Environment Western Sydney University Sydney New South Wales Australia; ^44^ Centre for Biodiversity and Environment Research, Department of Genetics, Evolution and Environment University College London London UK; ^45^ Sino‐French Institute for Earth System Science, College of Urban and Environmental Sciences Peking University Beijing China; ^46^ CREAF Campus Universitat Autònoma de Barcelona, Cerdanyola del Vallès Barcelona Catalonia Spain; ^47^ CSIC, Global Ecology Unit CREAF‐ CSIC‐UAB, Bellaterra Barcelona Catalonia Spain; ^48^ Institute of Carbon Neutrality, Sino‐French Institute for Earth System Science, College of Urban and Environmental Sciences Peking University Beijing China; ^49^ State Key Laboratory of Tibetan Plateau Earth System, Resources and Environment (TPESRE) Institute of Tibetan Plateau Research, Chinese Academy of Sciences Beijing China; ^50^ Environmental Futures University of Wollongong, Dharawal Country Wollongong New South Wales Australia; ^51^ Research Institute of Agriculture and Life Sciences Seoul National University Seoul South Korea; ^52^ Interdisciplinary Program in Landscape Architecture Seoul National University Seoul South Korea; ^53^ Integrated Major in Smart City Global Convergence Seoul National University Seoul South Korea; ^54^ Department of Landscape Architecture and Rural Systems Engineering Seoul National University Seoul South Korea; ^55^ Department of Ecology and Evolutionary Biology University of Toronto Toronto Ontario Canada; ^56^ College of Bioscience & Biotechnology Hunan Agricultural University Changsha PR China; ^57^ Hunan Engineering Laboratory of Miscanthus Ecological Applications Hunan Agricultural University Changsha PR China; ^58^ Yuelushan Laboratory Changsha China; ^59^ Institute of Biological and Environmental Sciences, School of Biological Sciences University of Aberdeen Aberdeen UK; ^60^ Institute of Chemistry University of São Paulo (USP) São Paulo São Paulo Brazil; ^61^ Faculty of Civil and Environmental Engineering Technion– Israel Institute of Technology Haifa Israel; ^62^ Grand Technion Energy Program Technion– Israel Institute of Technology Haifa Israel; ^63^ Climate Change Cluster University of Technology Sydney Ultimo New South Wales Australia; ^64^ KAUST Coral Restoration Initiative (KCRI) and Division of Biological and Environmental Science and Engineering (BESE) King Abdullah University of Science and Technology Thuwal Saudi Arabia; ^65^ Key Laboratory of Breeding Biotechnology and Sustainable Aquaculture Chinese Academy of Sciences Qingdao China; ^66^ Laboratory for Marine Biology and Biotechnology Qingdao Marine Science and Technology Center Qingdao China; ^67^ Shandong Province Key Laboratory of Experimental Marine Biology Institute of Oceanology, Chinese Academy of Sciences Qingdao China; ^68^ Division of Plant Sciences Research School of Biology, The Australian National University Canberra Australian Capital Territory Australia; ^69^ Department of Biology The University of Western Ontario London Ontario Canada; ^70^ Nicholas School of the Environment Duke University Durham North Carolina USA; ^71^ School of Biological Sciences and Institute for Climate and Carbon Neutrality The University of Hong Kong Pokfulam, Hong Kong China; ^72^ State Key Laboratory of Agrobiotechnology (CUHK) Shatin, Hong Kong China; ^73^ State Key Laboratory of Soil and Sustainable Agriculture Institute of Soil Science, Chinese Academy of Sciences Nanjing China

Professor Stephen P. Long, FRS (Steve) was born in London in 1950. His passion for plant science was the result of an inspiring schoolteacher, Ms. Muriel Hoskins, and recognition that new science and biotechnology were needed to address the famines being experienced by the Global South in the 1960s. As a result, he studied agricultural botany at Reading University, graduating with a B.Sc. in 1972. He then went on to earn a Ph.D. from Leeds University in 1976 for research that revealed that plants with C4 photosynthesis were naturally present in the UK and not limited to tropical regions. He added to this formal education while visiting Kenya and India as a teacher for the United Nations Environment Programme (UNEP) during the 1970s and 1980s. These formative experiences underpinned a research agenda that Steve would pursue with remarkable success for the rest of his career, that is, understanding and engineering photosynthesis to improve agricultural productivity, resilience and sustainability in the face of global environmental change. His landmark contributions included work on C4 photosynthesis, photosynthetic responses to both heat and cold, photosynthetic and productivity responses to atmospheric changes, photosynthetic productivity of biomass crops, cross‐scale modelling of photosynthesis and plant function, and engineering of photosynthesis to improve crop yield. Along the way, Steve consistently kept pace with the latest technologies, finding ways to leverage them to accelerate progress. This included use of *in silico* modelling, cutting‐edge methods for growing plants under global change treatments in the field, and myriad biotech approaches to crop engineering.

One key hallmark of Steve's career was his ability to build teams, both for research and scientific publishing. He established early examples of transdisciplinary research teams for plant science at the SoyFACE project, Energy Biosciences Institute (EBI), and Realizing Increased Photosynthetic Efficiency (RIPE) projects. These transdisciplinary teams attracted funding from industry, philanthropic and government sources that were distributed among national and international networks of collaborators. During this time, he met a number of former and current editors of *Global Change Biology*; his warmth and support from many interactions are fondly remembered by them.

He also kept his “finger on the pulse” of the research community when working as a journal editor for nine journals. This included being the Chief and Founding Editor of: *Global Change Biology*, *Global Change Biology Bioenergy*, and *in silico Plants*. At the time of his death, he had also just conceived and helped to launch *Global Change Biology Communications*. These journals created new opportunities for research communities to form on topics of special societal importance and at the intersection of traditionally siloed subdisciplines. For all these achievements, Steve was recognized with many awards and prizes, including honorary doctorates from Lancaster University in 2007 and the University of Essex in 2023. He was elected a Fellow of the Royal Society in 2013 and a member of the U.S. National Academy of Sciences in 2019. His expertise was recognized with invitations to provide briefings to President George W. Bush, Princess Anne, and Pope Benedict XVI. Maybe most significantly, Steve's impact continues to be amplified by those he recruited into plant science and trained. With great care for people at all career stages and boundless energy, he mentored more than 50 postdoctoral scientists and graduate students, many of whom went onto successful scientific careers in academia and industry.

Steve amazed his colleagues with his enthusiasm and knowledge of a wide range of fields, but his particular passions were to exploit his research to solve critical human problems, with bioenergy and photosynthetic enhancement being two areas of greatest interest. His bioenergy interests date to at least the 1990s and were linked to his desire to give people new tools to stop global warming. Steve developed large, well‐funded programmes geared to translate basic research advances into new applications. Steve joined the University of Illinois, Urbana‐Champaign from the University of Essex in 1999, where he established the first experimental dedicated bioenergy crop field trials in Illinois, and showed that Miscanthus could be produced successfully in Illinois conditions. That work, along with Steve's reputation and expertise, laid the foundation for the establishment of the BP‐funded Energy Biosciences Institute at the University of Illinois, in collaboration with UC Berkeley and Lawrence Livermore National Laboratory, the largest public‐private partnership that any university had established. Steve's vision developed into a strong interdisciplinary programme in advanced biofuels from cellulosic feedstocks at Illinois that has gone on to successfully transition to establish a DOE‐funded Bioenergy Research Center (Center for Advanced Bioenergy and Bioproducts Innovation, CABBI) at Illinois. He put the University of Illinois on the map of places leading the field in advanced biofuel research. From just a handful of faculty members who were working directly on biofuels at Illinois in 2007, there are now more than 60 staff that are engaged in CABBI. He has left an indelible mark on the University of Illinois and people that will carry forth his legacy.

Steve also laid the foundation for the “plants as factories” paradigm by leading teams that demonstrated how bioenergy crops like sugarcane could be transformed to produce oil in their stems and leaves as a feedstock for biofuel. But Steve was not content with these achievements. The Realizing Increased Photosynthetic Efficiency (RIPE) project, a Gates Foundation‐funded program, developed technologies to increase photosynthetic efficiency and revolutionized our understanding of photosynthesis and crop productivity, laying the foundation for sustainable agricultural innovation worldwide.

Steve was the Chief and Founding Editor of *Global Change Biology* and *GCB Bioenergy*. For the current and former editors of *Global Change Biology*, and for the wider community, the establishment of *Global Change Biology* was a pivotal moment in global change biological and ecological research. Until then, articles appeared in the range of zoological/microbiological/ecological/plant science publications—the establishment of *Global Change Biology* gave us a dedicated publication outlet devoted to what we were all realizing would become a major issue—global environmental change and its consequences. Steve had the goal for *Global Change Biology* to be the world's leading journal in the field and worked relentlessly to his last day to make that a reality. Through GCB Bioenergy, Steve inspired an interdisciplinary framework, where global environmental change intersected with agronomic, technological, economic and policy development for the emerging bioeconomy.

Scientifically, Steve consistently challenged us to look ahead, asking: ‘What's the next emergent topic in global change biology? Who should we invite to review it? What commentary should we write?’ He was ambitious, not for personal gain, but for the journal itself, driven by a vision that *Global Change Biology* should continuously push the boundaries of biological sciences in global change research.

After 30 years of development, *Global Change Biology* has grown into a family of journals, including *GCB Bioenergy*, and now *GCB Communications*, which not only reflects the journal's evolution but also Steve's commitment to mentoring the next generation. The strength of *Global Change Biology* lies in the diversity of disciplines centered on global change. Recognizing that many outstanding manuscripts were being rejected from *Global Change Biology* due to space limitations, evidence in itself of *Global Change Biology*'s success, he championed the formation of the new journal *Global Change Biology Communications*. True to Steve's nature, the journal has yet to publish its first issue but is already exceeding expectations for submissions.

Steve's leadership made serving on the editorial board of *Global Change Biology* a truly special experience for us as Editors. Early in the development of the journals, he invited emerging talented scientists in the field from multiple disciplines and countries to join him in the adventure, and since then, it has remained a firm objective of his leadership to raise the profile, representativeness, creativity and effectiveness of the editorial board. Many of us were thrilled to have published our PhD and Post‐Doctoral research papers in *Global Change Biology* or *GCB Bioenergy* before becoming editors. Our experience as Editors has been unique, largely because Steve fostered a genuine community among Editors. Steve showed a sincere interest in what was going on in our lives, both scientifically and personally, and this extended to his interactions with the superb team supporting *Global Change Biology* and *GCB Bioenergy*, and staff from the publishers, Wiley. Steve had a great sense of British humour that came through in his presentations, the Halloween parties he organized in his home, and meetings and daily interactions with him. A great example of this is the TED talk he gave on “Can we hack photosynthesis to feed the world?” https://www.ted.com/talks/steve_long_can_we_hack_photosynthesis_to_feed_the_world.

Steve was extremely supportive of junior colleagues, and placed great importance on interdisciplinary collaborations and in co‐locating researchers from different disciplines under the same roof to foster those collaborations. He championed early‐career researchers and supported women scientists to excel, and he enabled us to be true to ourselves and make a difference—to be brave. He showed, through example, that it is possible to achieve greatness while maintaining a healthy work‐life balance.

Steve had a remarkable ability to balance being the ‘chief’ of the editorial board, offering leadership, mentorship, and an authoritative voice when needed, while being exceptionally trusting in his team, always handing us the reins and backing us to make good and fair decisions. His openness to suggestions, his trust in colleagues, and his commitment to supporting and developing new editors have been central to the remarkable success of *Global Change Biology*.

Beyond his outstanding scientific insights, Steve possessed a rare ability to make science, and our *Global Change Biology* meetings, genuinely enjoyable. He masterfully orchestrated the journal, drawing on the diverse opinions of his colleagues to create a symphony where every instrument sounded its best. Always open to hearing different perspectives, and often very diplomatically guiding us toward a shared understanding, with kindness and wisdom that made him a truly exceptional people coordinator. Importantly, Steve always had our backs. Not a day went by where you felt alone as a *Global Change Biology* Editor. He ensured we were heard, protected, and integral to editorial decisions, fostering a strong sense of teamwork. As Editors, we genuinely enjoyed Steve's and each other's company, often laughing late into the night in Oxford pubs! Steve made *Global Change Biology* more than just a journal; he made it a family. Thanks to Steve, serving as Editors for *Global Change Biology* and *GCB Bioenergy*, has been a source of joy and pride for us.

Steve's absence will be deeply felt, and we will cherish his friendship and kindness on many levels. But his mentorship, humility, and dedication to excellence inspired all who had the opportunity to work with him. His legacy will continue to live on through the transformative journals he built, the generations of researchers he guided, and the scientific ideals he championed with passion and integrity. We will miss Steve—but we will do our best to continue his vision into the future.

**Figure:**
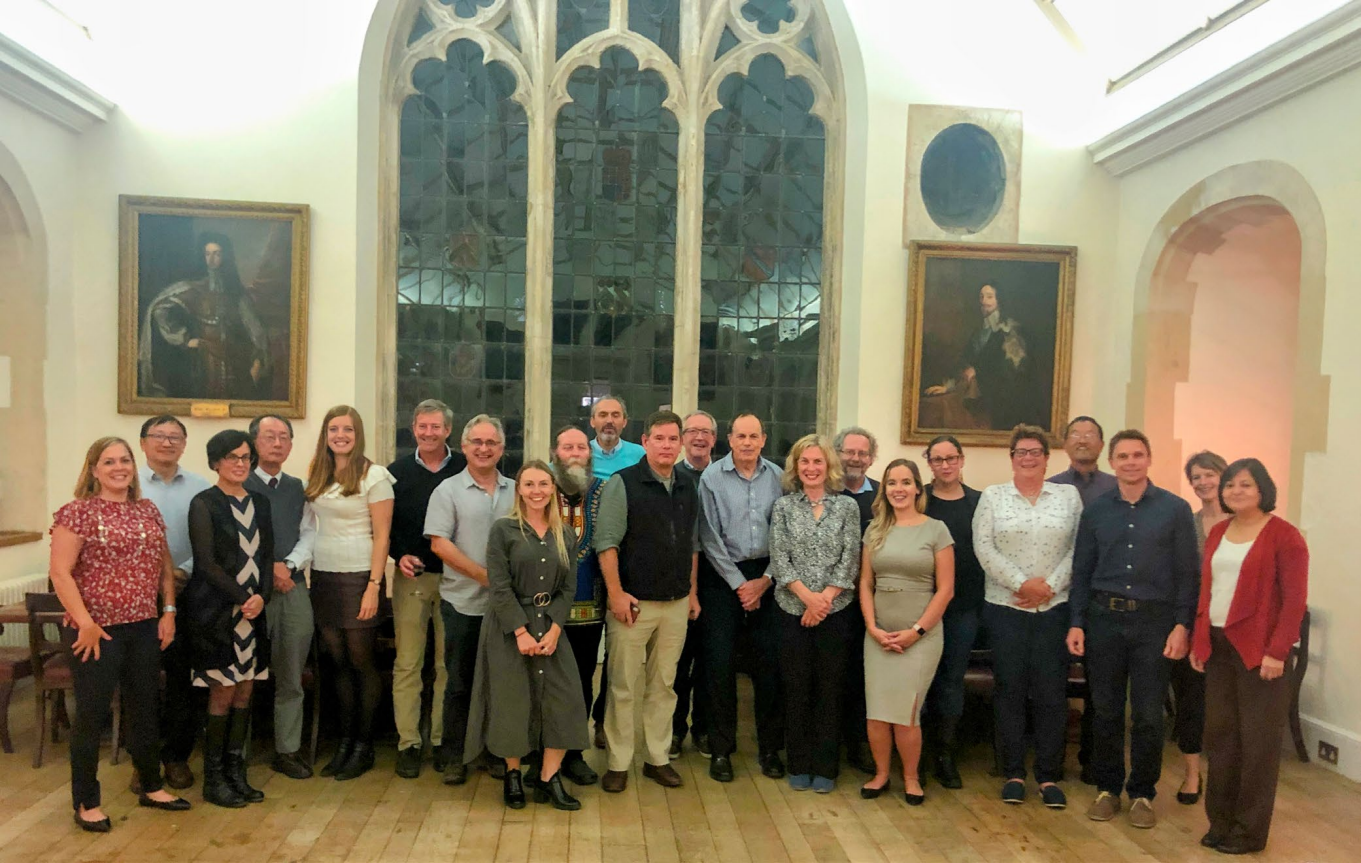
Global Change Biology Editors gathering in Oxford, England in 2019.

